# Is reverse cholesterol transport regulated by active cholesterol?

**DOI:** 10.1016/j.jlr.2023.100385

**Published:** 2023-05-09

**Authors:** Theodore L. Steck, Yvonne Lange

**Affiliations:** 1 Department of Biochemistry and Molecular Biology, University of Chicago, Chicago, IL, USA; 2 Department of Pathology, Rush University Medical Center, Chicago, IL, USA

**Keywords:** ABCA1, ABCG1, ApoA-I, HDL, homeostasis, LXR, regulation, reverse, SR-BI, 27-hydroxycholesterol

## Abstract

This review considers the hypothesis that a small portion of plasma membrane cholesterol regulates reverse cholesterol transport in coordination with overall cellular homeostasis. It appears that almost all of the plasma membrane cholesterol is held in stoichiometric complexes with bilayer phospholipids. The minor fraction of cholesterol that exceeds the complexation capacity of the phospholipids is called active cholesterol. It has an elevated chemical activity and circulates among the organelles. It also moves down its chemical activity gradient to plasma HDL, facilitated by the activity of ABCA1, ABCG1, and SR-BI. ABCA1 initiates this process by perturbing the organization of the plasma membrane bilayer, thereby priming its phospholipids for translocation to apoA-I to form nascent HDL. The active excess sterol and that activated by ABCA1 itself follow the phospholipids to the nascent HDL. ABCG1 similarly rearranges the bilayer and sends additional active cholesterol to nascent HDL, while SR-BI simply facilitates the equilibration of the active sterol between plasma membranes and plasma proteins. Active cholesterol also flows downhill to cytoplasmic membranes where it serves both as a feedback signal to homeostatic ER proteins and as the substrate for the synthesis of mitochondrial 27-hydroxycholesterol (27HC). 27HC binds the LXR and promotes the expression of the aforementioned transport proteins. 27HC-LXR also activates ABCA1 by competitively displacing its inhibitor, unliganded LXR. § Considerable indirect evidence suggests that active cholesterol serves as both a substrate and a feedback signal for reverse cholesterol transport. Direct tests of this novel hypothesis are proposed.

Cell cholesterol is held at its physiologic setpoint by a battery of molecular mechanisms (1). Excess cholesterol is transferred to acceptors in the plasma by the action of three plasma membrane proteins: ABCA1, ABCG1, and SR-BI (2, 3, 4). How this process of reverse cholesterol transport is coordinated with other homeostatic activities remains to be established. It seems that neither total cell cholesterol nor a particular organelle or protein orchestrates this complex process. Rather, it has been suggested that the marginal sterol that accumulates above the physiological setpoint of the cell provides an important feedback signal (1, 5). This excess fraction is called active cholesterol. We now examine the hypothesis that active cholesterol is mobilized and released by the activities of ABCA1, ABCG1, and SR-BI. Furthermore, we argue that, by feeding the synthesis of 27-hydroxycholesterol (27HC), active cholesterol also promotes the expression of the three aforementioned proteins and reverses the inhibition of ABCA1 by LXR.

We first describe the behavior of active cholesterol and then review literature concerning the three proteins and functions related to them. Direct evidence supporting our hypothesis is sparse. The presentation is therefore speculative; sometimes contentions are expressed without qualification to facilitate the flow of ideas. The argument is basic and general; it does not consider a variety of activities related to reverse cholesterol transport that do not bear directly on the premise such as the complex management of the body’s cholesterol by the liver (6, 7). A simplified representation of the hypothesis is presented in Fig. 1.

**Fig. 1:**
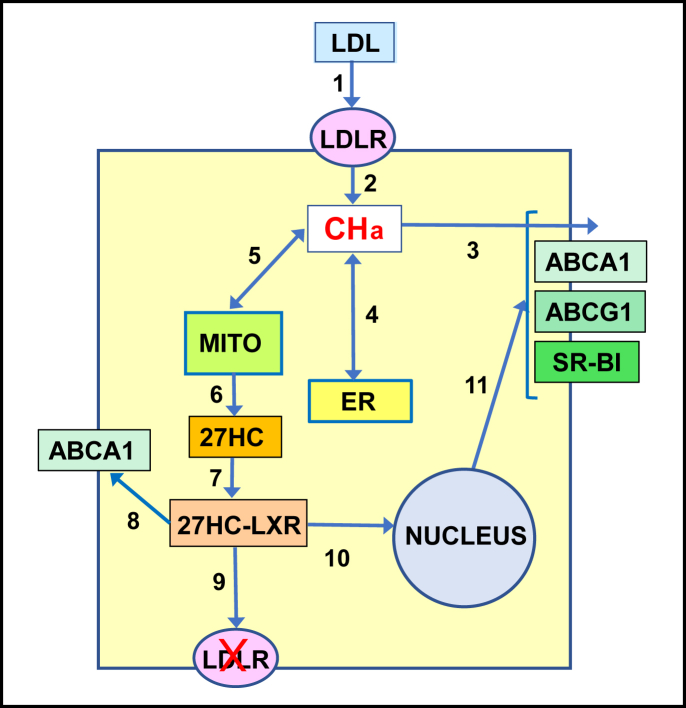
Hypothesis: Active cholesterol regulates reverse cholesterol transport, RCT (see text). An excess of cell cholesterol can arise at times, for example, from the uptake of low density lipoproteins (LDLs, arrow 1). Excess cholesterol is thermodynamically active, denoted as CHa (arrow 2). Three RCT proteins facilitate the release of active cholesterol down its chemical gradient to plasma acceptors (arrow 3). In addition, active cholesterol circulates through the cytoplasm and stimulates the feedback regulation of cholesterol homeostasis in the endoplasmic reticulum (arrow 4). It is also the substrate for the synthesis of 27-hydroxycholesterol in the mitochondria (arrows 5 and 6). The association of 27HC with LXR (arrow 7) directly activates ABCA1 (arrow 8), stimulates the clearance of LDL receptors (arrow 9), and promotes the expression of the three RCT proteins (arrows 10 and 11). These responses restore cell cholesterol to its physiologic setpoint.

## Active cholesterol

**Fig. 2:**
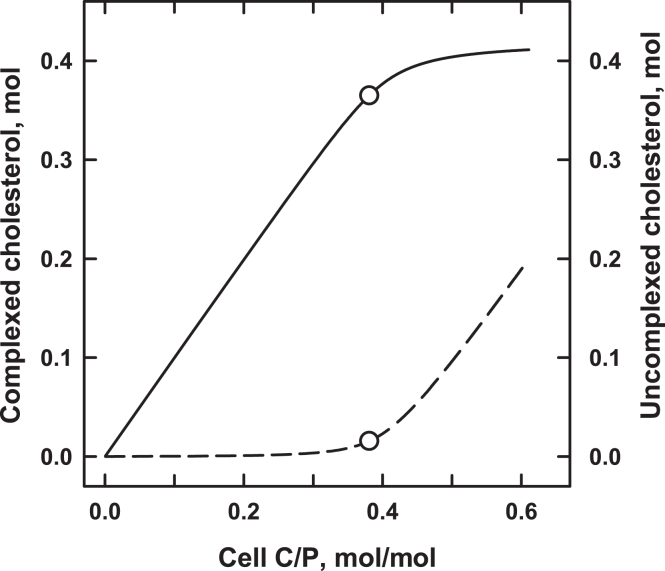
Uncomplexed cholesterol arises when the sterol exceeds the stoichiometric capacity of the membrane phospholipids. The curves are for an idealized cell in which the sterol is held in phospholipid complexes (solid line) or is uncomplexed (dashed line). The stoichiometric equivalence point of the plasma membrane occurs at a cell cholesterol of 0.38 mol cholesterol/mol phospholipid (circles). Several homeostatic feedback activities are stimulated by the active cholesterol arising at that threshold (1, 5, 11). This review examines the hypothesis that reverse cholesterol transport is among them.

It is a strong hypothesis that almost all the unesterified cholesterol in animal cell membranes is associated with phospholipids (1, 8). These sterol-phospholipid complexes have characteristic affinities and simple stoichiometries; typically, one cholesterol molecule is complexed with one or two phospholipids (1, 9, 10). Uncomplexed cholesterol accumulates rapidly above the stoichiometric equivalence point of the membrane phospholipids (Fig. 2). The uncomplexed sterol exceeding the capacity of the phospholipids has a greatly elevated chemical activity and is therefore called active cholesterol (8). Membrane cholesterol can also be activated by removing phospholipids and by adding various membrane-intercalating amphiphiles that displace it from its phospholipid complexes; conversely, active cholesterol can be sequestered by the addition of phospholipids (5, 12). Furthermore, as discussed later, ABCA1 and ABCG1 as well as scramblase appear to activate cholesterol by reorganizing the bilayer. Uncomplexed membrane cholesterol can associate with both exogenous and integral proteins (13, 14). In addition, ligands such as cytolysins can extract the sterol from its phospholipid complexes competitively (14). Thus, three dispositions of membrane cholesterol are relevant here: the sterol held in complexes with phospholipids; the sterol extractable from complexes; and the active sterol excess. The last two forms are available to ligands and, together, are called accessible cholesterol (14).

Active cholesterol spontaneously exits lipid bilayers to acceptors (15, 16, 17). Two mechanisms for this passive transfer have been proposed. The simpler of the two is aqueous diffusion (3, 18, 19, 20). Here, cholesterol molecules desorb from the membrane, typically with half-times of several hours; the released sterol can be captured by acceptors in the surrounding unstirred aqueous layer (21). An alternative mechanism postulates that cholesterol molecules do not readily desorb from membranes but can be rapidly captured in a two-step activation-collision process (4, 8, 20, 22, 23). Here, sterols continuously bob by thermal motion orthogonal to the bilayer surface (activation) where they are occasionally captured by aqueous acceptors (collision) (24, 25). The aqueous diffusion and activation-collision mechanisms are not mutually exclusive and can be difficult to distinguish experimentally (3, 23). (Consequently, transfers ascribed to aqueous diffusion may actually be driven by activation-collision.) In some cells, the unassisted transfer of active cholesterol to an acceptor can be relatively substantial (2, 3, 18, 21, 26, 27). As described later, other cell types employ reverse cholesterol transport proteins to facilitate the process.

Active cholesterol equilibrates down its chemical activity gradient throughout the cell on a time scale of several minutes facilitated by a battery of transport proteins (15, 16, 17, 28, 29). Plasma membrane phospholipids are avid for the sterol and are essentially fully complexed with ∼0.7–0.8 moles of cholesterol per mole phospholipid (5, 8, 9). Plasma membranes contain ∼90% of the cell cholesterol in cells such as human fibroblasts (1). In sharp contrast, the phospholipids in the endomembranes have very weak sterol affinity and little cholesterol, perhaps ∼10% of the total. There is ∼0.05 moles cholesterol per mole of phospholipid in the ER and <0.1 moles per mole in the endomembranes (1, 14). This is because regulatory proteins hold the level of endomembrane cholesterol at a sharp threshold far below the stoichiometric equivalence point of their phospholipids (1, 14). An important consequence is that excess plasma membrane cholesterol can flow to the impoverished endomembranes (4, 28, 30). The homeostatic effectors in the ER and mitochondria read the active excess in those compartments as a negative feedback signal and respond in order to maintain cell cholesterol at its physiologic setpoint (11).

## 27HC promotes reverse cholesterol transport

Active cholesterol is the substrate for the synthesis of 27HC by sterol 27-hydroxylase, and a small excess of cell sterol acutely drives the production of the oxysterol (31, 32). 27HC then signals the homeostatic effectors to reduce the excess (33, 34). In particular, the oxysterol is an allosteric activator of cholesterol esterification (35). It also inhibits sterol biosynthesis both by stimulating the downregulation of 3-hydroxy-3-methylglutaryl coenzyme A reductase and by suppressing the activation of SREBP-2 (36, 37). Furthermore, 27HC activates liver X receptor/retinoid X receptor (LXR/RXR) heterodimers which drive the expression of a battery of proteins that mediate the reduction of excess cell cholesterol (38, 39). Among these are ABCA1, ABCG1, and SR-BI (25, 40, 41). The LXR activated by 27HC also reduces the uptake of cholesterol by promoting the degradation of low density lipoprotein receptors (42). [A recent study presents a contrasting view of the role of 27HC (37).]

27HC also activates ABCA1 directly, as follows. Unliganded LXR**β** associates with ABCA1 and inhibits its function. 27HC bound to LXR**β** reverses this effect. That is, 27HC-LXR**β** outcompetes the unliganded LXRβ inhibitor for ABCA1, thereby activating it (43, 44).

As mentioned above, excess plasma membrane cholesterol sharply increases the active cholesterol in the ER where it elicits a variety of acute feedback responses (5, 11). Presumably, one of these responses is the production of 25-HC, another important homeostatic effector (37, 45). It has been argued that 25-HC, like 27HC, stimulates the expression of the proteins that mediate reverse cholesterol transport (46). However, this inference is not secure (37, 47).

## ABCA1 drives the release of plasma membrane phospholipids to APOA-I

ABCA1 belongs to a large family of ABC ATPases that transport diverse small molecules, especially lipids, across plasma membranes (48, 49, 50). The primary role of ABCA1 is to facilitate the exit of excess plasma membrane cholesterol for redistribution among the tissues as well as to the liver and intestine for enteric excretion (51). The mechanism of sterol transfer is complex and debated (7, 25, 27, 50, 52, 53, 54, 55, 56). Clearly, donor cells first generate an acceptor for the release of the sterol. This is nascent HDL: small phospholipid bilayer discs girded by apoA-I. One mechanism proposed for the creation of nascent HDL has the ATP-driven conformational cycle of ABCA1 transferring phospholipids from the outer leaflet of the plasma membrane bilayer laterally to the apoA-I associated with its extracellular domain (54, 56). ATP hydrolysis could serve a catalytic or an energetic function here, either speeding a downhill transfer reaction or driving it forward.

### Bilayer reorganization

We favor a different view of nascent HDL formation. In this scenario, ABCA1 uses the hydrolysis of ATP to reorganize the plasma membrane bilayer. This primes its phospholipids for downhill transfer to apoA-I (49, 57, 58, 59). One manifestation of this perturbation is the alteration of the lateral organization of the bilayer (49, 60, 61, 62). Another is the translocation of inner leaflet phospholipids such as phosphatidylserine and phosphatidylinositol 4,5-bisphosphate to the outer leaflet (27, 49, 59, 63, 64). ABCA1 is not a calcium-triggered scramblase; that is, not a conduit for the nonspecific exchange of phospholipids between the bilayer leaflets (65). Instead, it is often viewed as a floppase; that is, an ATP-powered transporter of inner leaflet lipid molecules to the outer leaflet (7, 50, 55, 56, 66, 67). As discussed shortly, we do not favor the floppase hypothesis. However, at this point, the mechanism by which ABCA1 reorganizes the bilayer is not understood.

### Transfer of phospholipids to ApoA-I

How then is nascent HDL constructed? It appears that ABCA1 promotes the association of apoA1 with the plasma membrane (49, 50, 52, 53, 68). In particular, the binding of the apoprotein to the extracellular domain of ABCA1 fosters its integration into the bilayer, perhaps by facilitating its partial unfolding (69, 70). The rearrangement of the bilayer by ABCA1 also facilitates apoprotein binding. For one thing, the altered bilayer presents acute local curvature that favors apoA-I binding (49, 71). In fact, the bending of the bilayer by the action of ABCA1 can instigate the release of membrane lipid particles in the absence of apoA-I (27, 70). Furthermore, the transfer of anionic phospholipids to the outer leaflet also promotes the association of the apoprotein (25, 59, 63). Finally, the disordering of the plasma membrane bilayer might itself favor apoA-I integration. On the other hand, the removal of outer leaflet phospholipids by ABCA1 is not needed to make room for the inner leaflet constituents, given that the protein causes phosphatidylserine to cross the bilayer in the absence of apoA-I (58, 72). [Like ABCA1, scramblase brings anionic phospholipids to the cell surface and alters membrane curvature (65). It would be interesting to know whether scramblase activity also promotes apoA-I binding and/or the release of lipid particles.]

### Elaboration of nascent HDL

ApoA-I extracts a few percent of the phospholipids in plasma membrane bilayers to form discoidal HDL particles. This can occur even in the absence of ABCA1 and cholesterol (73, 74, 75). One view is that apoA-I removes bits or domains of plasma membrane bilayer en bloc by a process called microsolubilization (49, 76, 77). Our hypothesis calls for a different mechanism: nascent HDL is assembled as the apoA-I draws plasma membrane phospholipid molecules down their chemical activity gradient (78, 79). The sterol follows, as discussed next. Such a mechanism can explain the selectivity observed among the transferred phospholipids (80, 81). It also accounts both for the low cholesterol/phospholipid ratio found in nascent HDL and for the variance of its cholesterol content with physiologic conditions (78, 82, 83).

## ABCA1 facilitates efflux of plasma membrane cholesterol to nascent HDL

The principal role of ABCA1 is to remove excess cell cholesterol (7, 25, 50, 84, 85). Accordingly, cholesterol enrichment stimulates ABCA1 activity (2, 82, 86). That active cholesterol is the form of the sterol released is suggested by the inhibition of the process by excess plasma membrane sphingomyelin (64, 87, 88, 89, 90). This is because the sphingomyelin holds cholesterol in tight complexes and thereby reduces its chemical activity (91). Conversely, sphingomyelinase liberates cholesterol for transport (91). [The ceramide generated by sphingomyelinase treatment also activates cholesterol directly by competitively displacing it from its phospholipid complexes (12). Ceramide also increases the abundance of ABCA1 at the cell surface, perhaps through the active cholesterol/27HC/LXR gene expression pathway described above (92).]

ABCA1 creates active cholesterol itself, presumably by liberating it from complexes. This is seen as an increased accessibility of the sterol to probes such as cholesterol oxidase and methyl-β-cyclodextrin when nascent HDL is not there to remove it (62, 93). ABCA1 also facilitates the association of perfringolysin O with the plasma membrane (67, 90, 94, 95, 96). The binding of such cytolysins has an accessible cholesterol threshold similar to that shown in Fig. 2 (14).

The efflux of cholesterol to nascent HDL can closely parallel that of the phospholipids (54, 74, 86, 97). However, the exit of the two lipids seems not to be directly coupled. Rather, the release of cholesterol follows that of the phospholipids in a two-step process: sequentially rather than simultaneously (7, 58, 78, 98, 99, 100, 101, 102, 103). How then does ABCA1 stimulate cholesterol export? One of two favored hypotheses is that ABCA1 is a cholesterol floppase (7, 50, 55, 67, 90, 94, 95, 96, 104). That is, ATP hydrolysis powers the translocation of the sterol from the cytoplasmic to the exoplasmic leaflet of the plasma membrane bilayer. High resolution cryo-electron microscopy points to a sterol pathway through the protein and an ATP-driven conformational cycle (55, 56, 66). This hypothesis predicts that ABCA1 generates a pool of active cholesterol in the outer leaflet for downhill release to nascent HDL. However, the transport of cholesterol molecules across the bilayer by ABCA1 has yet to be directly demonstrated. Furthermore, the ATPase activity of ABCA1 is only around one cycle per second in vitro. Could this slow pace maintain a transmembrane sterol activity gradient against its spontaneous ∼10 μs relaxation (23, 27, 105)? And would such a futile pump-leak cycle not consume an inordinate amount of metabolic energy in the process (105)? This problem would not arise if ABCA1 delivered the sterol directly to the nascent HDL acceptor. However, most of the active cholesterol released to nascent HDL is from the bilayer rather than from the ABCA1 (7). Furthermore, as mentioned above, ABCA1 can create a stable pool of active membrane cholesterol in the absence of apoA-I. Thus, our hypothesis does not favor the floppase mechanism.

An alternative to the floppase mechanism posits that the perturbation of the plasma membrane bilayer by ABCA1 raises the chemical activity of its cholesterol. [In an analogous case, phospholipid scrambling by scramblase boosts the accessibility of cholesterol at the erythrocyte surface (106).] Reorganization of the phospholipids by ABCA1 does not depend on cholesterol (58). The opposite is the case: it is the rearranged bilayer that stabilizes the active cholesterol (27). Furthermore, active cholesterol is not taken up by the apoA-I itself (78, 107). Rather, it is the phospholipid bilayer in the nascent HDL that accepts the sterol (3, 7, 27, 50, 93, 100, 101, 104). Presumably, this transfer is favored thermodynamically because the phospholipids in the nascent HDL are enriched in avid phosphatidylcholine and sphingomyelin and therefore have a higher affinity for cholesterol than the parent plasma membrane (49). A favorable chemical gradient is maintained by the subsequent esterification of the HDL cholesterol by LCAT (27).

Thus, the active cholesterol released to nascent HDL derives from three sources. The primary one is the sterol that accumulates at times beyond the stoichiometric capacity of the plasma membrane phospholipids. Second, ABCA1 removes phospholipids from the plasma membrane and leaves behind uncomplexed cholesterol primed for transfer to the nascent HDL. Third, ABCA1 activates cholesterol by reorganizing the plasma membrane bilayer. None of these three mechanisms involves cholesterol pumping by a sterol floppase. Rather, ABCA1 enables the passive flux of the sterol to the nascent HDL acceptor that it has generated.

## ABCG1

Like ABCA1, ABCG1 is expressed in response to excess cholesterol (32, 38, 108). Presumably, this is because active cholesterol feeds the synthesis of 27HC which then associates with LXR and activates the expression of these proteins (31). ABCG1 then enables the transfer of active cholesterol to the phospholipid bilayer in nascent HDL so as to further its maturation (25, 109, 110, 111). Evidence for the role of active cholesterol is that plasma membrane sphingomyelin inhibits ABCG1-mediated sterol release (62, 90, 112). Presumably, the sphingolipid forms complexes with active cholesterol that reduce its availability. This is a telling finding, given that sphingomyelin is an activator of ABCG1 (113, 114).

ABCG1 resembles ABCA1 in several ways. However, ABCG1 lacks the specialized extracellular domain by which apoA-I binds to ABCA1. Consequently, it facilitates the nonspecific exit of cholesterol to a variety of circulating acceptors (25, 115, 116, 117). ABCG1 is thought by some to be a floppase, utilizing ATP hydrolysis to drive sterol molecules from the cytoplasmic to the exoplasmic leaflet of the plasma membrane (67, 118). In fact, cryo-electron microscopy shows two cholesterol molecules and possibly a sphingomyelin within the transmembrane cavity of the protein (119, 120, 121). A sterol floppase could create a pool of active cholesterol in the outer leaflet that is primed for release to nascent HDL. However, such an active transport mechanism would struggle to maintain a trans-bilayer cholesterol activity gradient against its rapid back diffusion (24, 105).

We favor an alternative mechanism in which ABCG1, like ABCA1, perturbs the plasma membrane bilayer (3, 112, 113, 122). This premise is supported by the finding that ABCG1 promotes the efflux of phosphatidylcholine and sphingomyelin to plasma proteins (117, 122). The effect of reorganizing the bilayer would be to elevate the chemical activity of the sterol and promote its downhill flux to acceptors (3, 26, 62, 112, 123). One could imagine that, as suggested for ABCG5/G8, the cholesterol molecules activated by ABCG1 project from the plasma membrane bilayer surface and are captured by their collision with acceptors (8, 24). In addition, the preferential transport of phospholipids out of the plasma membrane bilayer would, of itself, leave uncomplexed active cholesterol behind and ready to follow. This cannot be the whole story, however, because ABCA1 and ABCG1 generate active cholesterol in the absence of an acceptor for the membrane phospholipid (26, 93).

## HOW do ABCA1 and ABCG1 affect ER cholesterol?

Multiple feedback mechanisms in the ER help to regulate the level of cell cholesterol, cued by the active cholesterol circulating through the cell (1, 5, 45). When apoA-I or nascent HDL is limited, both ABCA1 and ABCG1 send plasma membrane cholesterol to the ER (26, 87, 93, 112, 124). Thus, at least in vitro, nascent HDL and the ER act in parallel to reduce excess sterol homeostatically (98). The cholesterol transferred to the ER is taken from the inner leaflet of the plasma membrane bilayer (125). This finding suggests that ABCA1 and ABCG1 are not sterol floppases because, if they were, they would deplete the inner leaflet of cholesterol and divert it from the ER; however, the opposite is observed. [Some reports take a different view of this issue (90, 94).] That ABCA1 and ABCG1 increase the level of cholesterol in the ER adds to the evidence discussed above that they activate plasma membrane sterol.

## Does active cholesterol manage the disposition of ABCA1 and ABCG1?

ABCA1 and ABCG1 cycle between the plasma membrane and cytoplasmic organelles as they facilitate the release of excess cell cholesterol (7, 49, 126, 127). It is plausible that active cholesterol regulates this traffic in a homeostatic fashion. Supporting this conjecture is the finding that LXR promotes the redistribution of endosomal ABCG1 to the plasma membrane (116). Presumably, this effect is mediated by the 27HC derived from active cholesterol (31). Active cholesterol could play a role in other management functions as well (27). For example, it might promote the associations of ABCA1 and ABCG1 with caveolae and caveolin-1 that leads to cholesterol efflux (7, 128). A variety of other caveolae-mediated activities could similarly serve cholesterol homeostasis (5).

Active cholesterol could also impact the abundance and activity of ABCA1 and ABCG1 post-translationally (25, 84, 85). The cholesterol-binding sites on these proteins might be instrumental (55, 129, 130). In particular, ABCA1 and ABCG1 are turned over with half-times of a few hours (25, 85, 131). Inhibition of their proteolytic processing by excess cholesterol might be mediated by the active fraction. The turnover of ABCA1 and ABCG1 is also sensitive to their state of phosphorylation and to their association with calmodulin (131, 132, 133, 134, 135). Active cholesterol itself and/or the 27HC derived therefrom could modulate the effectors of these activities or the susceptibility of their targets in a homeostatic fashion.

## SR-BI

SR-BI is a plasma membrane protein, the expression of which is stimulated by excess cholesterol presumably by feeding the production of 27HC-LXR complexes (40, 41). Two of its many functions are relevant here (3, 104, 136). First, SR-BI binds HDL and mediates the transfer of its esterified cholesterol through the plasma membrane to the cytoplasm. Second, it mediates the bidirectional flux of active cholesterol down its chemical activity gradient between membranes and acceptors (27). In particular, SR-BI exchanges plasma membrane cholesterol with HDL and other lipoproteins (137). Furthermore, it plays an important role in cholesterol excretion, conducting the sterol from the plasma to liver and intestinal cells for delivery to acceptors in the bile and gut (138, 139, 140, 141).

A cavity within SR-BI might serve as a sterol pathway, as envisioned for ABC proteins (142). However, the protein lacks ATPase activity and should not raise the chemical activity of membrane cholesterol on its own. It is therefore of interest that it increases the binding of cytolysins to plasma membranes and their susceptibility to cholesterol oxidase (41, 137, 143, 144, 145). Perhaps this is because SR-BI boosts the activity of plasma membrane cholesterol by facilitating the uptake of the sterol from plasma lipoproteins (136, 145).

## Conclusions

We have argued that, in its role as a central coordinator of sterol homeostasis, active cholesterol serves both as a substrate for reverse cholesterol transport and as a feedback signal that regulates the expression and activity of the requisite proteins (Fig. 1). It appears that the energy-dependent reorganization of the plasma membrane bilayer by ABCA1 activates its phospholipids, priming them to flow downhill to apoA-I. This builds the sterol acceptor, nascent HDL. Excess cholesterol has a high chemical activity, and ABCA1, ABCG1, and SR-BI facilitate its downhill release from membranes to acceptors. These proteins also provide active cholesterol to the ER where it downregulates cholesterol accretion in a feedback fashion. Furthermore, as the substrate for 27HC synthesis, active cholesterol promotes the expression of the three “transport” proteins through LXR/RXR. 27HC also reverses the inhibition of ABCA1 by unliganded LXR. These feedback mechanisms null active cholesterol, returning cell sterol to its physiological setpoint. The pathways and molecular mechanisms by which the three proteins facilitate reverse cholesterol transport are still being clarified.

Given that the function of ABCA1 and ABCG1 is to clear excess cholesterol, why do these proteins also activate and release the sterol residing in phospholipid complexes (26, 93)? Perhaps doing so assists in the removal of the excess sterol. In particular, perturbation of the bilayer would increase the chemical gradient driving cholesterol transfer from the plasma membrane to the nascent HDL. Alternatively, cholesterol activation could be an inevitable consequence of the perturbation of bilayer organization by the ABC proteins. In any case, the resident sterol and phospholipids released by their activity would have to be replaced lest the plasma membrane be diminished.

Another hypothetical issue is that the activation of plasma membrane cholesterol could create a positive feedback loop that, unchecked, would lead to the runaway depletion of cell cholesterol. This could occur if the complexed cholesterol activated by the ABC proteins fed the formation of 27HC, thereby further stimulating the expression of ABCA1 and ABCG1 as well as directly increasing the activity of ABCA1. Presumably, such a regenerative cycle is countered physiologically. Perhaps, in vivo, the activated plasma membrane cholesterol is transferred to nascent HDL so rapidly that it does not feed 27HC production. In addition, strong feedback responses by the ER might preempt a vicious cycle (5, 11).

The hypothesis that active cholesterol regulates reverse cholesterol transport is in need of direct experimental support. Missing are complete cholesterol concentration-dependence curves for the relevant functions, such as illustrated in Fig. 2. Dose-response curves can be constructed using methyl-β-cyclodextrin to add and remove the sterol (11). Prediction: sharp thresholds will be obtained for the dependence of various RCT activities on the level of cholesterol as it exceeds the physiologic setpoint of the cell. We therefore suggest the determination of dose-response curves for the following activities using established methods:

• The unmediated transfer of plasma membrane cholesterol to nascent HDL and other acceptors (17, 146).
• The ABCA1- and ABCG1-facilitated transfer of cell cholesterol to nascent HDL (147).
• The SR-BI-facilitated transfer of cell cholesterol to acceptors (137).
• The binding of cholesterol to the reverse cholesterol transport proteins (129, 130).
• The synthesis of 27HC (31).
• The expression of the reverse cholesterol transport proteins (25, 41, 50).
• The turnover of the reverse cholesterol transport proteins (85, 131).
• The phosphorylation of the reverse cholesterol transport proteins (85, 133).
• The distribution of the reverse cholesterol transport proteins between the plasma membrane and endosomes (49, 116).
• The effect on the functions listed above of (a) membrane-intercalating amphiphiles that displace cholesterol from its complexes and thereby activate it; and (b) amphiphiles such as short-chain phospholipids that complex cholesterol and thereby suppress its activity (5, 12, 17).

If the basic premise is affirmed, it will be worth considering whether active cholesterol coordinates the particularly complex handling of cholesterol in hepatocytes and enterocytes. These cells utilize not only ABCA1, ABCG1, and SR-BI but other sterol transporters and effectors to maintain the balance of their cholesterol along with that of the whole body (6, 148, 149).

## Conflict of interest

The authors declare that they have no known competing financial interests or personal relationships that could have appeared to influence the work reported in this paper.
